# Reduced human-biting preferences of the African malaria vectors *Anopheles arabiensis* and *Anopheles gambiae* in an urban context: controlled, competitive host-preference experiments in Tanzania

**DOI:** 10.1186/s12936-020-03495-z

**Published:** 2020-11-20

**Authors:** Yeromin P. Mlacha, Prosper P. Chaki, Athuman Muhili, Dennis J. Massue, Marcel Tanner, Silas Majambere, Gerry F. Killen, Nicodem J. Govella

**Affiliations:** 1 Ecological Sciences Department, Ifakara Health Institute, Environmental Health, Kiko Avenue, P.O. Box 78373, Mikocheni, Dar es Salaam, United Republic of Tanzania; 2 Swiss Tropical and Public Health Institute, Basel, Switzerland; 3 University of Basel, Basel, Switzerland; 4 The Pan-African Mosquito Control Association (PAMCA), KEMRI Headquarters, Mbagathi Road, Nairobi, 54840-00200 Nairobi Kenya; 5 Univerity of Dar Es Salaam, Mbeya College of Health and Allied Sciences, P.O. Box 608, Mbeya, United Republic of Tanzania; 6 Amani Research Centre, National Institute for Medical Research, P.O. Box 81, Muheza-Tanga, United Republic of Tanzania; 7 Liverpool School of Tropical Medicine, Vector Biology Department, Pembroke Place, Liverpool, L3 5QA UK; 8 School of Biological, Earth & Environmental Sciences and Environmental Research Institute, University College Cork, Cork, Republic of Ireland; 9 The Nelson Mandela, African Institution of Science and Technology, The School of Life Science and Bio-Engineering (LISBE), P.O.BOX 447, Tengeru Arusha, United Republic of Tanzania

**Keywords:** Malaria, Vector, *Anopheles*, Host preferences, Residual transmission, Entomological surveillance, Tanzania

## Abstract

**Background:**

Host preference is a critical determinant of human exposure to vector-borne infections and the impact of vector control interventions. Widespread use of long-lasting insecticide-treated nets (LLINs) and indoor residual spraying (IRS) across sub-Saharan Africa, which protect humans against mosquitoes, may select for altered host preference traits of malaria vectors over the long term. Here, the host preferences of *Anopheles arabiensis* and *Anopheles gambiae *sensu stricto (s.s.) were experimentally assessed in the field, using direct host-preference assays in two distinct ecological settings in Tanzania.

**Methods:**

Eight Ifakara Tent Trap (ITT), four baited with humans and four with bovine calves, were simultaneously used to catch malaria vectors in open field sites in urban and rural Tanzania. The numbers of mosquitoes collected in human-baited traps *versus* calf-baited traps were used to estimate human feeding preference for each site's vector species.

**Results:**

The estimated proportion [95% confidence interval (CI)] of mosquitoes attacking humans rather than cattle was 0.60 [0.40, 0.77] for *An. arabiensis* in the rural setting and 0.61 [0.32, 0.85] for *An. gambiae s.s.* in the urban setting, indicating no preference for either host in both cases (P = 0.32 and 0.46, respectively) and no difference in preference between the two (Odds Ratio (OR) [95%] = 0.95 [0.30, 3.01], *P* = 0.924). However, only a quarter of *An. arabiensis* in the urban setting attacked humans (0.25 [0.09, 0.53]), indicating a preference for cattle that approached significance (P = 0.08). Indeed, urban *An. arabiensis* were less likely to attack humans rather than cattle when compared to the same species in the rural setting (OR [95%] = 0.21 [0.05, 0.91], *P* = 0.037).

**Conclusion:**

Urban *An. arabiensis* had a stronger preference for cattle than the rural population and urban *An. gambiae s.s.* showed no clear preference for either humans or cattle. In the urban setting, both species exhibited stronger tendencies to attack cattle than previous studies of the same species in rural contexts. Cattle keeping may, therefore, particularly limit the impact of human-targeted vector control interventions in Dar es Salaam and perhaps in other African towns and cities.

## Background

Apart from the distributions of bites between inside and outsides the houses and at different times of the night [1, 2], what mosquitoes feed upon critically determines the choice and impact of human-targeted vector control interventions [3–8]. For example, both historical and recent reports [9–14] show that the widespread use of long-lasting insecticide-treated nets (LLNs) or indoor residual spraying (IRS), which directly target humans or houses they live in, strongly suppressed or virtually eliminated the population of the main malaria vectors *Anopheles gambiae *sensu stricto (s.s.) and *Anopheles funestus s.s*. These two species preferentially feed upon human blood across sub-Saharan Africa (SSA) [10, 11, 15–18]. Beyond Africa, *Anopheles darlingi* was eliminated in British Guiana following three years of IRS with DDT [19]. This same species appears to have disappeared in Suriname in response to the scale-up of LLINs [20]. These vectors are highly vulnerable to insecticide-based interventions for protecting humans because these species rely heavily upon human blood for their survival [7, 19, 21–23].

While *Anopheles arabiensis* is commonly known to exhibit flexible host-feeding, switching biting between humans and domestic animals [24–27], recent evidence suggests that even the historically most inflexible human-feeding mosquito species in Africa, *An. funestus *s.s. can now attack non-human hosts, specifically cattle [24, 28]. This newly observed behavioural plasticity allows the mosquito to evade human-targeted insecticide-based interventions by allowing it to access safer alternative blood sources [29, 30]. This behaviour may help vector species sustain its population and contribute to residual malaria transmission by evading fatal contact with existing front-line interventions [6, 31, 32].

Inherent *host preference* is an innate behavioural trait of a mosquito population that is assessed in the field by allowing mosquitoes to freely select between two or more different host species experimentally presented in equal numbers simultaneously. Host choice, however, is a more complex function of both host preference and the availability of different host species that can be accessed locally and is assessed by surveying the sources of mosquito bloodmeals collected after they have fed [33, 34]. However, because the host choices exhibited by any given mosquito population can vary across spatial scales of only a few metres (e.g., in a cattle shed *versus* the house nearby), experimentally-controlled host preference measurements are a more reliable means of making direct comparisons between populations. Despite its critical importance as a metric to inform the selection of impact vector control interventions, there remains a paucity of data on vector host preference and its potential change over time.

Here, the inherent host attack preferences of *An. arabiensis* and *An. gambiae s.s.* only was assessed in two distinct ecological settings (urban *versus* rural) in Tanzania. A competitive preference experimentally-controlled assay, baited with either a human or calf, was simultaneously presented to malaria vectors. This study focused only on these two vector species because they are both important primary malaria vectors across Tanzania and elsewhere in Africa. Other, mostly secondary, malaria vector species were caught in insufficient numbers to be reliably assessed.

## Methods

### Study sites

This study was conducted at two different Tanzania regions: the urban Dar es Salaam and the rural village within the Kilombero valley in the Morogoro region. Dar es Salaam is the largest City of Tanzania, situated at 6° 51′S, 39° 18′E along the Indian Ocean with an estimate of 5 million people according to the national census of 2012 [35]. A detailed description of the study area has been previously published elsewhere [36, 37]. The main malaria vectors are *An. gambiae s.s.* and *An. arabiensis,* but *Anopheles merus* and *An. funestus s.s.* are also available, though existing in very low numbers throughout the year [38]. *Anopheles gambiae s.s.,* which is often regarded as the most anthropophagic vector (rely feeding heavily upon human blood), feeds predominantly in the middle of the night [36, 39]. In contrast, its sibling species, *An. arabiensis,* which is commonly referred to as zoophagic (prefers feeding on cattle) mosquito throughout SSA [22], starts actively feeding in the early evening and mainly outdoors, time which coincides with the period when most residents of this city are still outside [36, 39]. This overlaps overtime, and outdoor space between mosquito and human activity potentially increases the risk of human exposure to malaria transmission, which cannot be effectively addressed by using indoor-targeted interventions such as LLINs [39]. During this study, human *Plasmodium falciparum* malaria infection was around 10% among residents in all age groups [37], and with the strong reduction in malaria vectors densities of *An. gambiae* complex and *An. funestus* group [40]. This was achieved due to the scaling-up of larvicides [41] and LLINs [36, 37]. The scaling-up of larvicides and LLINs coincided spontaneously with the wide use of window screening across the city of Dar es Salaam [40]. The average annual rainfall ranging from 800 to 1300 mm with a 25ºC annual temperature [42].

The second study site was at Kilombero valley, Lupiro village (8°23′03.8″ S, 36°40′26.7″ E), which is located 40 km south of Ifakara town within the Kilombero Valley, south-eastern Tanzania [43]. The detail of an area can be found elsewhere [13, 43]. The area is located at 300 m above sea level on the floodplains of Kilombero valley. The average annual rainfall ranges between 1200 to 1800 mm between December to May, and the temperature is recorded at ranges from 20 to 32.6 °C. The most resident lives on subsistence farming of rice, fishing, and sparse livestock keeping. *An. arabiensis* and *An. funestus* group are the primary malaria vectors in the area, but the latter exist in relatively very low numbers throughout the year [12]. The historically-important malaria vector *An. gambiae s.s.* had been virtually eliminated, following the widespread use of LLINs [13].

### Experimental design

**Fig. 1:**
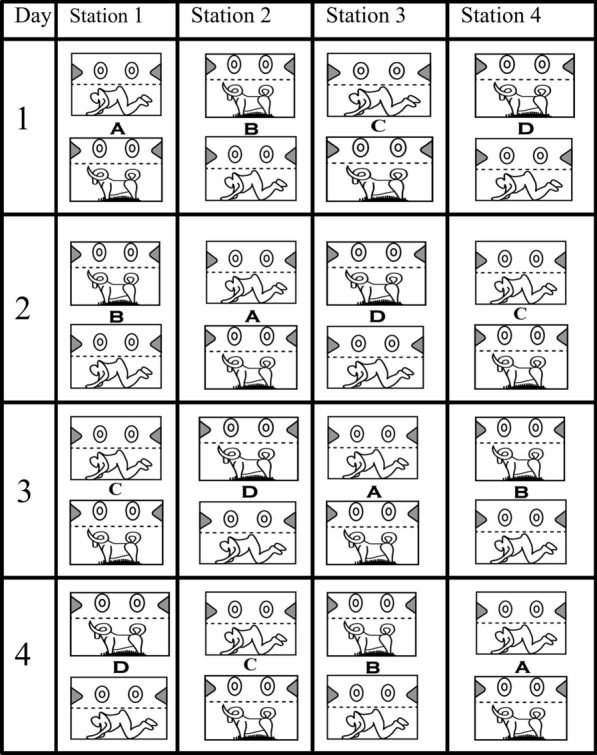
The schematic illustration of a typical 4 × 4 Latin square experimental design with one complete round of experimentation through four mosquito-capturing stations in the field area. The dashed line indicates a screen bisecting the upper and lower part of the trap, which protects volunteers from being exposed to mosquito bites. The ring and the funnel shape on the side illustrate the mosquito entry point

Eight Ifakara Tent Trap version C (ITT-C) [39] baited with either humans or calves were simultaneously used to catch wild malaria vectors in urban Dar es Salaam and rural Kilombero Valley. In each site, an open field ground measuring more than 500 m long was selected. Four (human *versus* calf) pairing catching stations, spaced about 50 m apart, were established within these field grounds. Within each pair, the host was spaced 5 m apart, allowing for a competitive host preference assay. A Latin square design involving the movement of trap-host combinations between positions was implemented to minimize possible biases associated with each position and natural variations in individual hosts' attractiveness to mosquitoes [44, 45]. Each pair was rotated after each experimental night through four stations. Four nights were required to make a complete round of experimentation (Fig. 1). After each round of four nights, the actual human volunteers and calves were replaced. The calf within each ITT-C was tethered to lure the mosquito entry inside the trap. Each morning, calves were taken out of the tent for daily grazing. There was no exchange of host between traps (calf-baited versus human-baited) because it was not acceptable to expect human participants to sleep in traps soiled by a calf. Trapping was conducted from 19:00 h to 06:00 h, and trapped mosquitoes were emptied from the trap every morning using a mouth aspirator. The details on how to empty mosquitoes inside the ITT-C can be found in the previous article [39]. In urban Dar es Salaam, 104 (60 nights between May to August 2009 and 44 nights between March and June 2010), experimental nights were conducted. In rural Kilombero Valley, only 16 nights (from August to September 2010) was conducted. It took longer in Urban Dar es Salaam due to the limited number of malaria vector densities.

### Mosquito identification

Every morning, trapped adult mosquitoes from each trap were collected by mouth aspirator, placed in a respective paper cup prior labelled according to the host, and killed using chloroform. Morphological identification was conducted based on the keys of Gillies and Coetzee [46]. All collected *An. gambiae *sensu lato (s.l.) were stored individually in Eppendorf tubes (1.5 ml) with silica gel desiccant and cotton before transport for Polymerase chain reaction (PCR) assay for species identification. The field-collected data were recorded and linked with laboratory results using the designated forms adapted from Kiware et al*.* [47].

### Statistical analysis

Statistical analyses were carried out using the R statistical software version 3.6.1, augmented with the *matrix*, *lattice,* and *lme4* packages. To test the effect of species-specific on attacking human host, only PCR confirmed individuals from the *An. gambiae* complex (*An. gambiae s.s.* and *An. arabiensis*) were used. Because the response variable for each species is binary (that is, an individual mosquito can only attack a single host at a time and not both), a Generalized Linear Mixed Effect Models (GLMMs) [48], using binomial distribution and logit link function, was applied. The proportion of mosquitoes caught attacking humans was treated as the response variable, with a variable combination of PCR confirmed species and sites as a fixed effect. The experimental night and stations were fitted as a random effect. The model was run first without fitting an intercept so that the absolute proportion of mosquitoes attacking the human for each species and from each site can be estimated and compared. This was followed by fitting models that included intercept to obtain the contrast in human feeding preference between species with *An. gambiae s.s*. in urban Dar es Salaam treated as a reference species in the model. This detailed statistical analysis on the effect of species on the propensity of attacking upon human host species was restricted to *An. arabiensis* and *An. gambiae s.s.*, partly because of their importance in driving malaria transmission in these settings, and their number captured was sufficient to detect the effect.

## Results

### Species composition

In urban Dar es Salaam, 197,155 mosquitoes were collected. 42,929 (21.8%) and 154,226 (78.2%) mosquitoes were collected from human and calf baited traps, respectively. The taxonomic group of mosquito collected included: *An. gambiae *s.l. (n = 97, 0.05%), *Anopheles coustani* (n = 2,144, 1.1%), *Culex* spp. (n = 192,836, 97.8%), *Mansonia* spp. (n = 1633, 0.8%) and *Coquillettidia* spp. (n = 460, 0.2%). All *An. gambiae s.l*. were subjected for PCR test, and 88 (88/97, 91%) specimens successfully amplified. Of which, 25 (28%) were *An. gambiae s.s*. and 63 (72%) *An. arabiensis.*

In rural Kilombero Valley, 41,876 mosquitoes were collected. 22,093 (53.0%) and 19,783 (47.2%) mosquitoes were collected from human and calf baited traps respectively. The taxonomic group of mosquito collected included: *An. gambiae s.l.* (n = 334, 0.8%), *An. funestus* group (n = 6, 0.01%), *An. coustani* (n = 185, 0.44%), *Anopheles ziemanni* (n = 31, 0.07%), *Culex* spp (n = 9539, 22.8%), *Mansonia* spp. (n = 31,749, 75.8%) and *Coquillettidia spp.* (n = 32, 0.08%). All *An. gambiae s.l.* were again subjected for PCR test, and all successful amplified specimens 313 (94%), confirmed to be *An. arabiensis*.

**Fig. 2:**
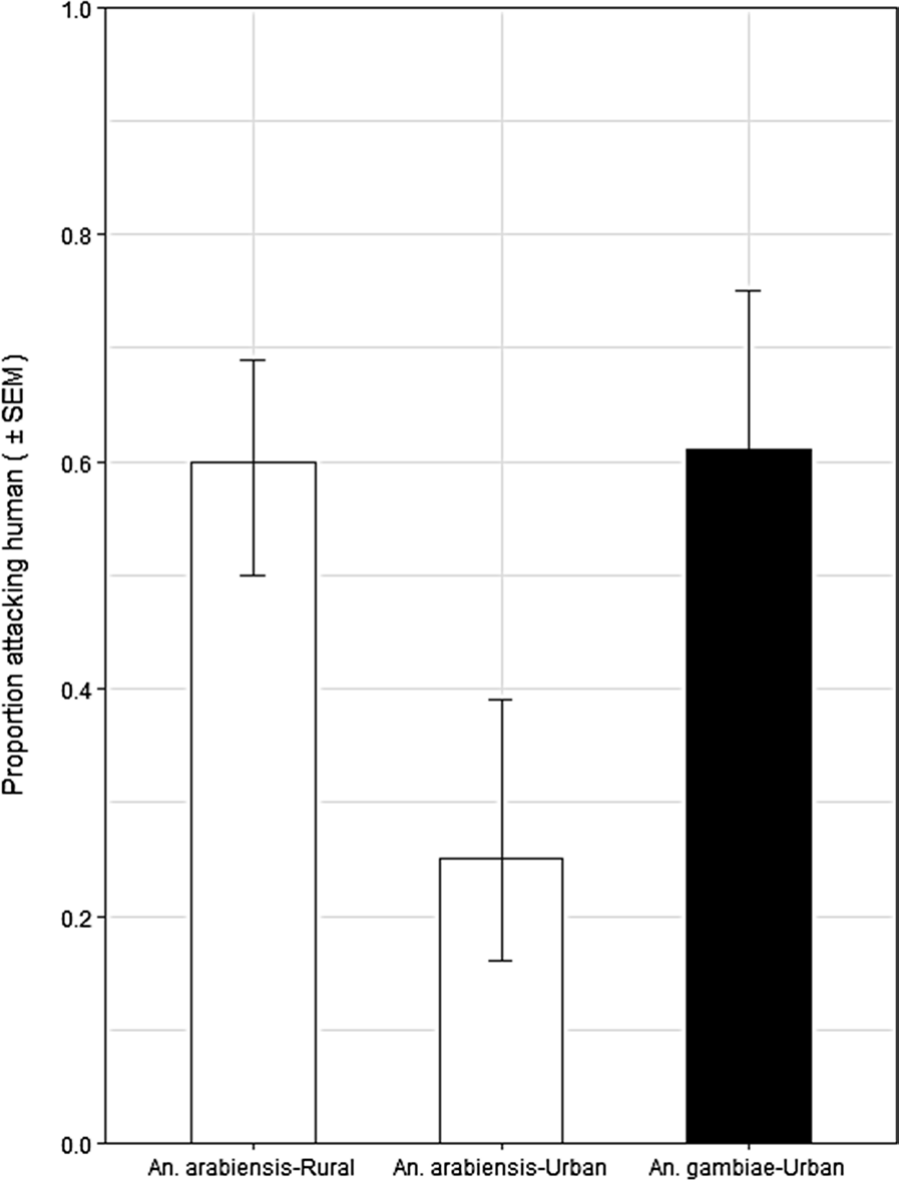
The proportion estimates (mean and standard error) attacking humans by the *An. gambiae s.s* and *An. arabiensis* captured in urban Dar es Salaam and rural Kilombero Valley

Based on the logistic model fitting to these data, the estimated proportion [95% confidence interval (CI)] of mosquitoes attacking humans rather than cattle was 0.60 [0.40, 0.77] for *An. arabiensis* in the rural setting and 0.61 [0.32, 0.85] for *An. gambiae s.s*. in the urban setting (Fig. 2), indicating no preference for either host in both cases (P = 0.32 and 0.46), respectively, with no evidence for any difference in preference between the two (Odds ratio (OR) [95%] = 0.95 [0.30, 3.01], *P* = 0.924)). However, only a quarter of *An. arabiensis* in the urban setting attacked humans (0.25 [0.09, 0.54]; Fig. 2), indicating a preference for cattle that approached significance (P = 0.081). Indeed, *An. arabiensis* in the urban setting were less likely to attack humans rather than cattle when compared to the same species in the rural setting (OR [95%] = 0.21 [0.05, 0.91], *P* = 0.037).

## Discussion

**Fig. 3:**
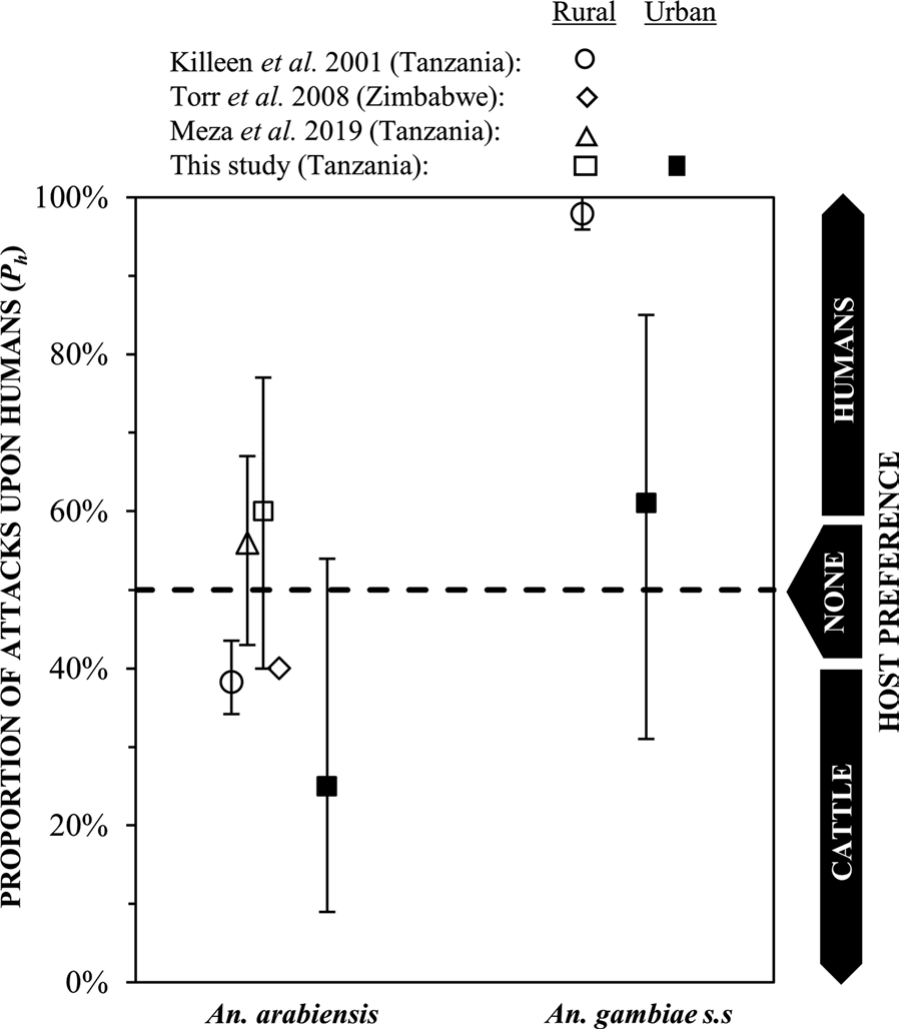
Previously estimated proportion of attacks on humans versus cattle (*P*_*h*_)) when offered a direct choice between one of each host species (mean and 95% confidence intervals, for *An. arabiensis* in rural Tanzania (data extracted from Fig. 4 in [24], and rural Zimbabwe (data extracted from Fig. 7 in [26]), and the estimated proportion of attacks on humans for *An. arabiensis* and *An. gambiae s.s*. obtained from historical records in the rural coastal region of Tanzania [21] compared to those obtained by this study in Kilombero, rural southern Tanzania, and Dar es Salaam, urban coastal Tanzania. The estimated proportion of attacks on humans (*P*_*h*_) from historical records were derived from modelling analysis of the relative availability of humans versus cattle (λ) models: *P*_*h*_ = 1/(1 + λ) [21]

The findings indicate variation in the preference for feeding upon humans rather than cattle between two populations of *An. arabiensis*, in urban Dar es Salaam, and rural Kilombero. These observations become more interesting and seem to suggest an effect of urban environments on both *An. arabiensis* and *An. gambiae s.s.*, compared with preceding studies that also measured host preference through carefully controlled experiments. The rural Tanzanian *An. arabiensis* population studied here had no strong preference for humans or cattle. Indeed these results compared particularly well with those of Meza et al*.* [24] (Fig. 3), which also used juvenile cattle with relatively low biomass, therefore, similar levels of attractiveness [49]. However, in urban Dar es Salaam, *An. arabiensis* appeared to exhibit a strong preference for cattle over humans and significantly different from the same species in rural Kilombero over approximately the same period (Figs. 2 and 3). Also unexpectedly, *An. gambiae s.s.* collected in Dar es Salaam, lacked its notoriously strong preference for humans compared with equivalent indices derived from a previous study of the same species in rural Tanzania [21]. It appears that both siblings species have a stronger preference for non-human hosts in this urban context than in previously reported studies of rural populations of the same species [21, 24, 26] (Fig. 3).

The flexible feeding behaviour exhibited by the *An. arabiensis* in rural Kilombero is consistent with that reported by previous studies from the same setting [24] and beyond [26] that employed similarly direct, experimentally-controlled, host attack preference measurements but used different capture methods. It is also reassuring that fitting host preference and availability models to historical blood meal host choice data for the same species across entire villages [21, 50] yields similar indirect estimates, indicating only a slight preference for cattle (Fig. 3) even though such natural herds are dominated by larger adult cattle that may be reasonably expected to be more attractive [49]. Indeed the Torr et al*.* [26] direct host preference experiments using electric grids similar to Meza et al*.* [24], which also used adult cattle, yield almost identical estimates to these indirectly inferred from modelling analyses, confirming a slight preference of rural *An. arabiensis* for fully-grown cattle over humans. Such biologically and methodologically plausible triangulation of results from such different studies with such different methods suggests that the experimental approach applied here, including the first use of ITT-C [39] for experimental host preference studies, provides reliable and readily comparable indices of host preference. Therefore, it is reasonable to interpret the findings that *An. arabiensis* had a stronger preference for cattle in urban Dar es Salaam than in rural Kilombero or any previous studies population of the same species (Fig. 3) at face value.

It is also telling that a similar, and perhaps more surprising, the pattern was observed for the notoriously anthropophagic [6, 27, 34, 51] *An. *gambiae s.s. compared with a previous study of the same species in a rural Tanzanian context (Fig. 3). The lack of a clear preference for humans over cattle by An gambiae s.s. in this contemporary urban context contrasts starkly with historical records from Segera, only 258 km away from Dar es Salaam [21]. This unusually flexible feeding behaviour for An. gambiae s.s. in Dar es Salaam may also contribute to the persistence of this species in this settings, unlike other nearby ecological settings where it was virtually eliminated [11, 15], following widespread use of LLIN [11–13, 15]. The increasingly widespread use of LLINs [52], and high coverage of house window screening in urban Dar es Salaam [40], which limit safe access of mosquitoes to human blood, may have forced this species to develop a strategy which enables them to evade personal target protective interventions for humans by exploiting animal blood whenever they can find it.

Urban Dar es Salaam generally has fewer cattle than Kilombero, and probably in most other rural settings. It is, therefore, interesting that *An. arabiensis* now appears to have a stronger preference for feeding on cattle and perhaps on other non-human hosts that were not assessed here. It will be important to investigate whether the two populations are genetically distinct or not [53–55]. This may be especially important following the recent surge of interest in genetic manipulation approaches for malaria vector control [56]**.** Regardless of the underlying basis for this apparent trend towards greater zoophagy in both vector species in Dar es Salaam, on the one hand, it will limit the impacts of existing malaria vector control interventions like LLINs and mosquito-proofed window screening. On the other hand, it may provide opportunities for complementary approaches like veterinary insecticide treatments for livestock [6, 7, 57–60].

While this study was quite limited in terms of scale and sample size, it does raise some important questions that merit consideration beyond Dar es Salaam and Tanzania. Urbanization is known to influence host preferences in other mosquito taxa [61], and similar effects to those reported here might also occur in other African settings where *An. gambiae s.s.* and *An. arabiensis* continue to mediate malaria transmission, despite widespread use of LLINs [54]. Indeed, it is notable that few experimentally controlled host preference studies could be found to populate Fig. 3, despite the vital role that this trait plays in malaria transmission and control. Therefore, this finding strongly encourages more widespread measurement of mosquito feeding preferences across a diversity of ecological settings through routine programmatic surveillance [62]. This may help inform the selection and evaluation of complementary vector control interventions, ideally in an ecologically stratified manner.

## Conclusions

Urban *An. arabiensis* had a stronger preference for cattle than the rural population in this or previous studies. Furthermore, the urban *An. gambiae s.s.* assessed here had a weaker preference for humans over cattle than reported by a previous study of the same species in a nearby rural context. Cattle keeping may limit the impact of human-targeted vector control interventions in Dar es Salaam, and perhaps in other African towns and cities. Generalization of mosquito species host preferences across broad geographies or assuming that they may remain static traits may be misleading with respect to the selection of effective vector control interventions. Therefore, the characterization of vector feeding preferences across distinct ecological settings is recommended as a critical component of routine programmatic surveillance to inform the effective design, selection, implementation, and assessment of complementary new vector control interventions, ideally on an ecologically stratified basis.

## Data Availability

The Ifakara Health Institute, on behalf of the United Republic of Tanzania, owns all data. Data can be shared upon reasonable request in line with the Ifakara Health Institute’s data management and sharing policy.

## Abbreviations

ITT: Ifakara Tent Trap (C type)
LLINs: Long-lasting insecticides treated nets
IRS: Indoor residual spray
DDT: Dichlorodiphenyltrichloroethane
GLMM: Generalized linear mixed effect model
PCR: Polymerase chain reaction
CI: Confidence interval
SSA: Sub-Saharan Africa
